# Competency-based training and supervision in group problem management plus in the Kurdistan region of Iraq

**DOI:** 10.3389/fpsyt.2026.1824107

**Published:** 2026-06-15

**Authors:** Tekoshar Aram, Leticia Silvela, Peter Ventevogel

**Affiliations:** 1 Department of Clinical Psychology, Koya University, Erbil, Kurdistan Region, Iraq; 2 Research and Contextualization Department, Kurd Therapy, Erbil, Kurdistan Region, Iraq; 3 Community-based Protection Unit, United Nations High Commissioner for Refugees, Erbil, Kurdistan Region, Iraq; 4 Public Health Unit, United Nations High Commissioner for Refugees, Geneva, Switzerland

**Keywords:** competency-based assessment, EQUIP (ensuring quality in psychosocial support and mental health care) platform, evaluation, group problem management plus (gPM+), Kurdistan region of Iraq, monitoring and evaluation, training

## Abstract

**Background:**

Group Problem Management Plus (gPM+) is a scalable psychological intervention designed to be delivered by non-specialist mental health workers and has been widely implemented in humanitarian contexts. As part of the ongoing process of integrating refugees into routine national care systems, competency-based training and supervision were implemented in Duhok, within the Kurdistan Region of Iraq. Twelve participants from the Directorate General of Health (DoH), working in Syrian refugee camps, took part in a seven-day training programme, followed by supportive supervision over a six-month period. The programme aimed to enhance participants’ skills in delivering the group intervention to individuals experiencing psychological distress.

**Methods:**

The training programme consisted of a seven-day (56 hours), structured in-person course combining theoretical input, demonstration, and intensive role-play practice, followed by a six-month supervision (30 hours) phase that included regular group supervision sessions and individual on-site coaching focused on real-life implementation challenges. Individual and group-level competency improvements were assessed using the EQUIP (Ensuring Quality in Psychosocial Support and Mental Health Care) methodology, which provides a structured approach to monitoring and tracking participants’ progress in facilitation and intervention skills, enabling trainers to identify skill gaps and tailor supervision sessions effectively. This was supplemented by qualitative evaluations from both participants and trainers.

**Results:**

Participants showed notable reductions in harmful behaviours and improvements in competencies. Supervision sessions and role-playing exercises further reinforced learning and skill application. Participants also reported increased confidence, with feedback highlighting the value of problem-solving strategies and coping techniques.

**Conclusions:**

EQUIP proved effective in monitoring and guiding improvements among non-specialist staff delivering psychological interventions. The EQUIP platform may serve as a valuable tool for strengthening mental health and psychosocial support (MHPSS) training and interventions, and for facilitating the integration of programmes developed in humanitarian contexts into routine systems of care in low-resource settings.

## 1 Introduction

Problem Management Plus (PM+) is a scalable psychological intervention developed by the World Health Organization (WHO), available in both individual (PM+) and group (gPM+) formats, to support individuals impaired by distress in communities exposed to adversity, particularly in low-resource and humanitarian settings (1, 2). It is designed for delivery by non-specialist workers, equipping them with skills to manage psychological distress and to improve functioning. Over the last decade, PM+ has proven effective, leading to significant reductions in psychological distress and symptoms of anxiety and depression (3–5). A substantial part of the research on PM+ has been conducted with Syrian refugees, including in Jordan and Turkey (6–8) as part of the STRENGTHS consortium that assessed effectiveness, cost-effectiveness, and scale-up potential of PM+ with Syrian refugees across eight countries in Europe and the Middle East (9, 10). In Iraq, research conducted with internally displaced persons showed that PM+ can be delivered effectively and is highly acceptable to the Iraqi population (11).

Since 2018, United Nations High Commissioner for Refugees (UNHCR) has introduced scalable psychological interventions such as PM+, either as part of health and protection programmes or as standalone programmes, to support the mental health and psychosocial well-being of refugees and internally displaced persons (12). In Lebanon, UNHCR’s group PM+ programme, delivered by refugee outreach volunteers to Syrian refugees, showed significant improvements, including enhanced psychological well-being, improved functioning, and reduced symptoms of depression (13). In Iraq, UNHCR translated the manual for individual PM+ into the Kurdish Badini language (14) and introduced the method on a small scale with Kurdish-Syrian refugees in Duhok (15). Earlier local clinical research in Duhok demonstrated the effectiveness of trauma-focused psychotherapeutic approaches, such as Narrative Exposure Therapy, among refugees living in camps, highlighting both the extent of trauma exposure and the feasibility of scalable evidence-based interventions in this context (16, 17). These prior experiences provide a valuable foundation for scaling up structured, low-intensity models like PM+ delivered through non-specialist workers in the Kurdistan Region of Iraq.

To enhance the quality of PM+ delivery by non-specialist workers, competency-based training and supervision are essential to ensure that trainees demonstrate the practical skills required for effective delivery in low-resource settings (18). Supervision is often an overlooked component in service delivery for displaced people (19), while the need for structured quality assurance is supported by studies on supportive supervision in diverse humanitarian settings (20, 21). This is further corroborated by an external evaluation of the group PM+ programme delivered by Syrian refugee outreach volunteers in Lebanon, which concluded that careful selection, extensive training, and supervision were crucial to successful implementation. The report recommends enhancing the training and supervision of volunteers through competency-based tools to monitor helpers’ skill levels and provide timely feedback (13).

EQUIP (Ensuring Quality in Psychosocial and Mental Health Care) is a comprehensive package of tools developed by the World Health Organization (WHO) and United Nations Children’s Fund (UNICEF) to support competency-based assessment, training, and supervision of people delivering mental health and psychosocial support (MHPSS), particularly in low-resource and humanitarian settings (22). The online EQUIP platform enables users to conduct competency-based assessments throughout training and supervision for scalable psychological interventions, including gPM+ (23). Role plays are a key component of skill development, with trainees practising the delivery of psychological support in realistic situations while being observed by trainers who provide structured feedback using checklists. Over the past few years, the EQUIP methodology has been introduced across a wide range of settings to train, supervise, and assess providers delivering various psychological and mental health interventions (24), including PM+ (25). In a multi-site evaluation, the EQUIP approach was found to help trainees gain confidence and improve their skills, with both trainees and trainers reporting that role plays and feedback were highly valuable for learning (26). Recent evidence from a pilot study with refugees trained as PM+ providers in Switzerland confirms that EQUIP tools can successfully identify trainee strengths, highlight areas for improvement, and generate targeted feedback, further validating the potential of EQUIP to enhance mental health care quality across diverse contexts (27).

While this body of research highlights the importance of competency-based assessments in training and supervision, such approaches are still not routinely used in humanitarian and refugee programmes (28, 29). This is also the case in Iraq, which has hosted around 300,000 Syrian refugees and asylum-seekers for more than a decade (30). Most reside in the three governorates of the Kurdistan Region of Iraq, living in nine refugee camps and in urban settings. In the early years of the Syrian conflict, UNHCR and other humanitarian partners provided essential services through non-governmental organisations. UNHCR aims to progressively integrate refugees into national systems, which in Iraq has seen a planned reduction in camp based parallel services, as access to government supported national services has increased. During the Global Refugee Forum 2023, the Government of Iraq pledged to continue to be enable refugees to access public services, for such as health and education (31). Access to mental health and psychosocial support services were more complex to transfer due to the overall limited capacity of governmental mental health services in the Kurdistan Region of Iraq (32). UNHCR has therefore gradually phased out the direct provision of MHPSS services through NGO partners, shifting towards capacity-building within national health services in close collaboration with the Directorate General of Health (DoH) (33, 34). As part of this handover, a capacity-building programme was organised in 2024 by UNHCR and DoH, with the aim of training non-specialist DoH staff working in Syrian refugee camps to develop competency in delivering gPM+. The training programme was officially supported by the Directorate of Health and formed part of a broader strategy to integrate scalable psychological interventions into routine services. However, the ability to implement this strategy was constrained by budget allocations, which have severely affected programmes for mental health and psychosocial support (35, 36).

This article presents an evaluation of the training programme, incorporating continuous competency evaluations via the EQUIP platform, test results, and feedback from participants.

The study aims to: 1) measure changes in competencies among non-specialist mental health staff who participated in a gPM+ training programme, and 2) assess the utility of the EQUIP platform in facilitating competency-based assessments and its applicability for training within a routine health care setting in a middle-income country.

It was hypothesized that participants would demonstrate progressive improvements in competency levels over time, reflected in reduced harmful behaviours and increased use of basic and advanced helping skills, and that the EQUIP platform would effectively support identification of skill gaps and guide targeted supervision.

## 2 Methodology

### 2.1 Participants

The training programme included 12 non-specialist mental health workers (seven female, five male) from the Directorate of Health (DoH) in Duhok who were working as outreach workers, social workers, or psychosocial counsellors in refugee camps in Duhok governorate, in partnership with UNHCR. Participants were selected in coordination with the Directorate General of Health based on their roles in service delivery within refugee camps and their availability to participate in the full training and supervision programme; inclusion criteria focused on current engagement in psychosocial or community-based services. Eight participants held degrees in educational psychology. In the Iraqi context, educational psychology programmes primarily focus on learning processes and educational development and typically do not include formal clinical training or supervised counselling practice. In terms of reach, the training engaged 12 non-specialist DoH staff working across four refugee camps in Duhok, representing a subset of approximately 50 eligible staff involved in service delivery.

### 2.2 Capacity building

The programme consisted of 56 hours of classroom training and 30 hours of supervision delivered over a six-month period. It included five group supervision sessions and two individual supervision sessions per trainee. The training focused on developing practical skills for managing psychological distress. The training followed the standard gPM+ curriculum and was structured into sequential modules combining theoretical input, demonstration, and practice. Core components included active listening, use of open-ended questioning, structured problem-solving (including distinguishing between solvable and unsolvable problems), behavioural activation, stress management techniques such as breathing exercises, and core group facilitation competencies. Each theoretical session was followed by structured role-play exercises, during which participants practised the specific skills introduced in that module. Multiple role plays were conducted for each core strategy, allowing different participants to practise the same skills repeatedly. Peer feedback was encouraged to reinforce learning and reflection.

#### 2.2.1 Adaptation

Case examples, role plays, and training materials were culturally and linguistically adapted to reflect realistic social roles, gender norms, and typical interpersonal dynamics in the local context (e.g., family hierarchies and community responses to distress). Emotional expression and response styles were adapted to culturally appropriate norms, such as the indirect expression of distress and avoidance of shame-based topics. Local Kurdish (Kirmanji and Badini) idioms of distress, such as “breaking backbone” (şikandina piştê - پشت شکان) and “heart burn” (şewitandina dil - دڵ سوتان) were acknowledged and integrated into discussions to improve relevance and participant engagement (37). The training also emphasised fostering self-confidence and a sense of self-efficacy among trainees.

#### 2.2.2 Role plays

Role plays were central to the training methodology, providing participants with the opportunity to practise and refine their gPM+ facilitation skills. During these exercises, participants received competency assessment tools to evaluate the role-play performance of their peers based on specific skills relevant to the gPM+ intervention. This approach to peer feedback and group evaluation was designed to reinforce learning and improve competency in delivering the intervention. During the supervision sessions, role-play themes were selected based on EQUIP results and recommendations, focusing on areas that needed improvement, whether related to knowledge or group facilitation competencies.

#### 2.2.3 Supervision

A total of five group supervision sessions were conducted, along with two individual supervision sessions per participant at their field locations. Supervision was provided by the trainer, a licensed psychotherapist with experience in practising gPM+ and training and supervising non-specialists, along with co-facilitators, including two UNHCR staff and a mental health supervisor from DoH. Each supervision session focused on real-world application of gPM+ techniques and addressing any challenges participants faced in implementing the intervention. During each individual supervision session, group dynamics techniques were assessed by the supervisor, and the participants’ skills and knowledge were evaluated. In the group supervision sessions, challenges were discussed among trainees, and positive outcomes and success stories were shared. Based on EQUIP competency assessment results, specific topics were selected for role plays in each supervision session. Participants who required additional support were given the opportunity to practise these topics during role plays.

### 2.3 Assessment and evaluation tools

#### 2.3.1 Assessment of essential competencies

Two tools for competency-based assessment were used for the pre-, post-, and follow-up assessments:

1. The Group Facilitation Skills (GroupACT) tool (38), which evaluates group facilitation across several competency areas including establishing group guidelines, ensuring fair participation, fostering empathy, addressing logistical barriers, and facilitating collaborative problem-solving (39).
2. The PM+ Competencies Tool (40), which assesses facilitators across 12 specific competency areas required for delivering PM+, such as recognizing solvable and unsolvable problems, brainstorming and selecting solutions, creating action plans, and evaluating their outcomes (41).

Each competency domain in both tools is scored across four levels, representing a spectrum of performance. Assessments were conducted through observation of standardised role-plays, in which participants demonstrated their skills in simulated scenarios. Performance was evaluated using structured EQUIP competency checklists. Assessments were conducted by trained facilitators and supervisors who were familiar with the EQUIP framework. The four levels are defined as follows:

1. Level 1 (Harmful behaviours): Practitioner demonstrates actions that are unhelpful or potentially harmful to the client, such as breaking confidentiality, making promises, and blaming the client.
2. Level 2 (Some basic skills): Practitioner demonstrates some basic helping skills, but not all are applied consistently. Examples include active listening, open-ended questions, summarising, and showing empathy.
3. Level 3 (All basic skills): Practitioner consistently applies all basic helping skills appropriately.
4. Level 4 (All basic and some advanced skills): Practitioner demonstrates all basic skills and integrates some advanced helping techniques, such as validating emotional responses and asking the client to reflect on empathetic statements.

These tools were administered via the EQUIP online platform. Each participant’s competencies were evaluated five times during the training and supervision period, from the initial sessions to the final supervision. Assessments were conducted at the beginning of the training, immediately after the training, and at three points during the supervision sessions. This approach enabled continuous monitoring of areas requiring improvement and each participant’s progress over time. Further details are provided in **Table 1**.

**Table 1:** Methods of data collection.

Tool used	Purpose	Measure	Frequency
GroupACT and PM+ competency tool via EQUIP	Track participant’s progress in facilitation and intervention skills	Ordinal data using a four-level scoring system: use a four-level scoring system: Level 1: Unhelpful or potentially harmful behaviours. Level 2: Basic skills are not demonstrated or are incomplete. Level 3: All basic helping skills are demonstrated. Level 4: Both basic and advanced helping skills are demonstrated.	5 times: Pre, Post, and 3 Follow-ups
Self-efficacy dartboard	Participants’ self-assessment of their knowledge and skills.	Descriptive data: Proximity to the centre indicates higher confidence, while marks further from the centre reflect lower self-efficacy.	Before and after the training
Training feedback: structured feedback questionnaire	Gather participant’s feedback on training content, delivery, and areas for improvement	- Quantitative: Ordinal scale (1 = least satisfied, 5 = most satisfied). - Qualitative: Idiographic (individualized) data.	Post-training
Trainer’s feedback	To observe the participant’s behaviour and progress during the exercise and to tailor the sessions and provide feedback	Qualitative: descriptive and subjective data through notes gathered during the training and supervision sessions.	Continuously during the training (role plays) and the supervision sessions

#### 2.3.2 Self-reported confidence

Additionally, participants completed a brief self-report dartboard assessment before and after the training. This assessment measured participants’ confidence across various gPM+ skills. The centre of the board represented 100% confidence, while the outer edge represented 0% confidence. Participants placed dots on the board to indicate their confidence level for each skill, with dots closer to the centre indicating higher confidence and those toward the edge indicated lower confidence.

#### 2.3.3 Feedback from participants

The feedback assessment tool consisted of a series of questions evaluating participants’ perceptions of the training’s content, delivery, and impact on their skills and knowledge, using a five-point scale from 1 (strongly disagree) to 5 (strongly agree). Qualitative data were collected through three open-ended questions at the end of the training: 1) What did you find most useful in this training? 2) What was least useful in this training? 3) What suggestions do you have to improve the training for future participants? The responses were then analysed to identify common themes. These qualitative data were primarily used to capture participant feedback and contextualise findings at the end of the training, rather than to serve as a primary analytical component.

#### 2.3.4 Observations by trainer and supervisors during supervision

The trainers observed participants’ behaviour and progress during the training and supervision sessions and maintained detailed notes. These observations were discussed and organised to document the training process and identify areas for improvement.

### 2.4 Data analysis

Quantitative data from the EQUIP competency assessments were analysed using descriptive statistics, given the small sample size and the exploratory, practice-oriented nature of the study. Frequencies were calculated to describe the number of participants demonstrating each competency level at each assessment point. Mean scores were computed across competency domains to examine overall changes in harmful behaviours and progression in helping skills over time. Where appropriate, per-participant averages were also calculated to illustrate individual-level trends across the training and supervision phases.

Self-reported confidence data were analysed descriptively by comparing the distribution of responses between pre- and post-training assessments, with attention to shifts in proximity to the centre of the confidence scale.

Training feedback questionnaire data were summarised using descriptive statistics, including mean scores for each Likert-scale item, to assess participant satisfaction and perceived training effectiveness.

Qualitative data from open-ended feedback questions and trainer observation notes were limited in volume and were therefore analysed using a structured descriptive approach. Responses were reviewed and grouped based on similarity of content and repeated ideas. These summaries were used to capture common participant experiences, perceived benefits, and suggestions for improvement. Qualitative information was used to complement and contextualise the quantitative findings rather than to generate formal qualitative themes.

To further situate the findings within an implementation science perspective, selected elements of the RE-AIM framework were retrospectively applied to this programme, looking at the reach, effectiveness, adoption, implementation, and maintenance (42).

## 3 Results

### 3.1 Implementation outcomes: competency development

This study primarily reports on implementation-related outcomes, including competency development, feasibility of training delivery, and participant acceptability. The outcomes presented reflect changes in provider-level skills, behaviours, and perceived confidence following training and supervision. The study was not designed to assess clinical effectiveness or client-level outcomes, and therefore findings should be interpreted in terms of implementation and capacity-building rather than intervention impact.

#### 3.1.1 Problem management competencies results

**Figure 1:**
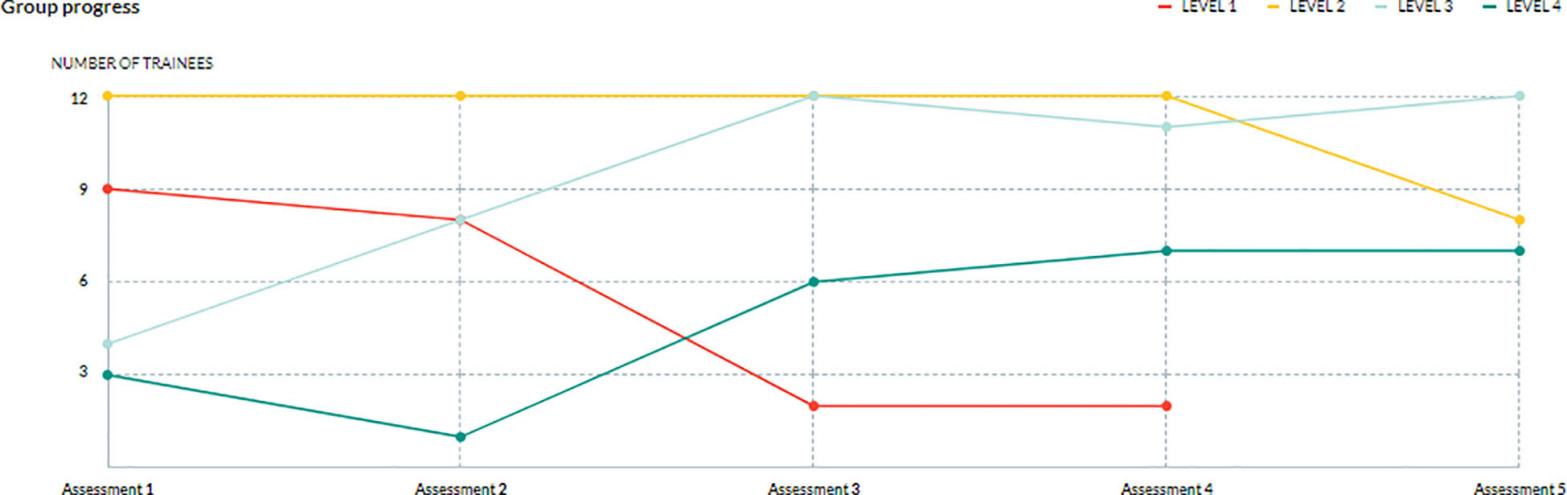
Group progress in problem management competency skills. LEGEND: Level 1: Any unhelpful behaviour. Level 2: Some but not all basic helping skills. Level 3: All basic helping skills. Level 4: All basic helping skills plus at least one advanced skill.

Results are presented using both the number of participants demonstrating each competency level and the mean number of behaviours per participant, in order to capture both group-level trends and changes in the intensity of behaviours over time. Initially, nine out of 12 participants (75%) exhibited at least one harmful behaviour during the first assessment. As shown in **Figure 1**, there was a consistent downward trend in harmful behaviours across assessment points, with all participants reaching zero harmful behaviours by the final assessment, indicating substantial improvement over time. This number decreased to eight participants in the second assessment (conducted immediately after the training) and further declined during subsequent sessions, ultimately reaching zero in the final assessment (**Figure 1**). When examining the total number of harmful behaviours separately from participant counts, a reduction was observed from 50 instances across 9 participants at baseline to 20 instances across 8 participants immediately post-training.

**Figure 2:**
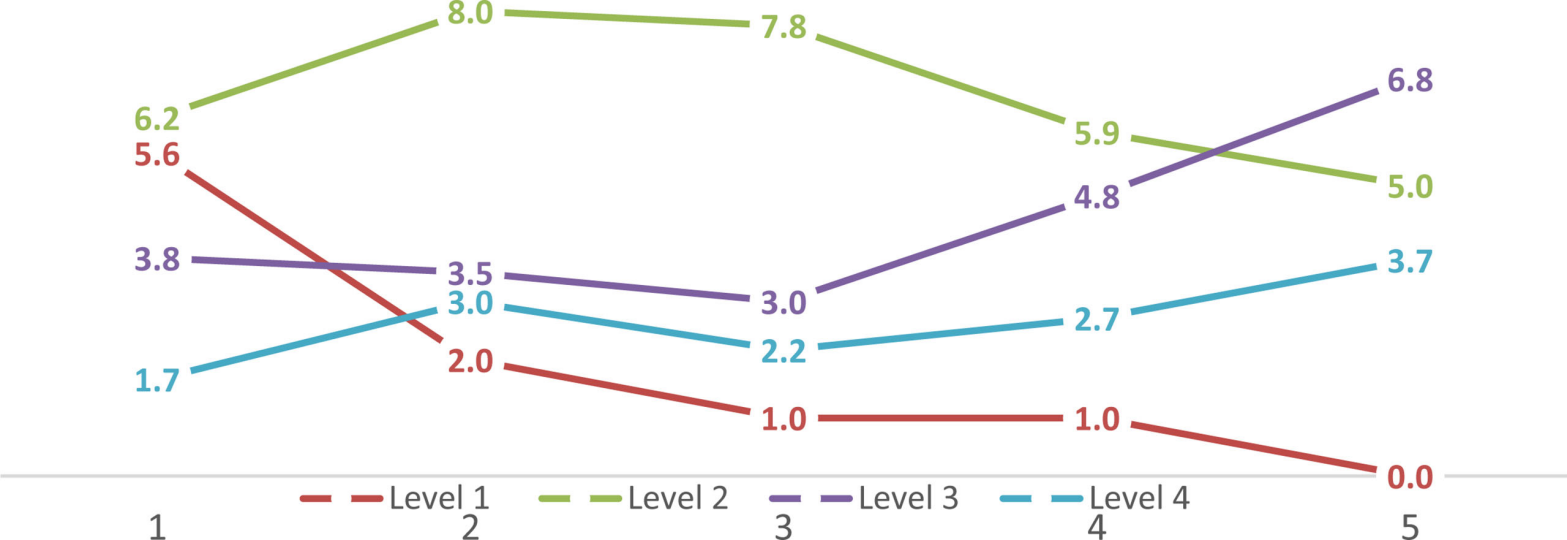
Number of problem management competency skills/behaviours per participant (mean) at each level.

This corresponds to a reduction in the mean number of harmful behaviours per participant from 5.6 in the initial assessment to 2.0 in the second assessment. The follow-up assessments conducted during the supervision sessions showed continued reduction in harmful behaviours, with a corresponding increase in both basic and advanced helping skills (**Figure 2**).

#### 3.1.2 Group facilitation skills results

Further data analysis showed a reduction in the number of participants displaying harmful behaviours, decreasing from nine in the initial assessment to seven in the post-training assessment, followed by continued reductions during the follow-up sessions, eventually reaching zero. This trend was accompanied by a gradual shift toward higher competency levels, with increasing numbers of participants demonstrating consistent use of basic and advanced facilitation skills over time.

**Figure 3:**
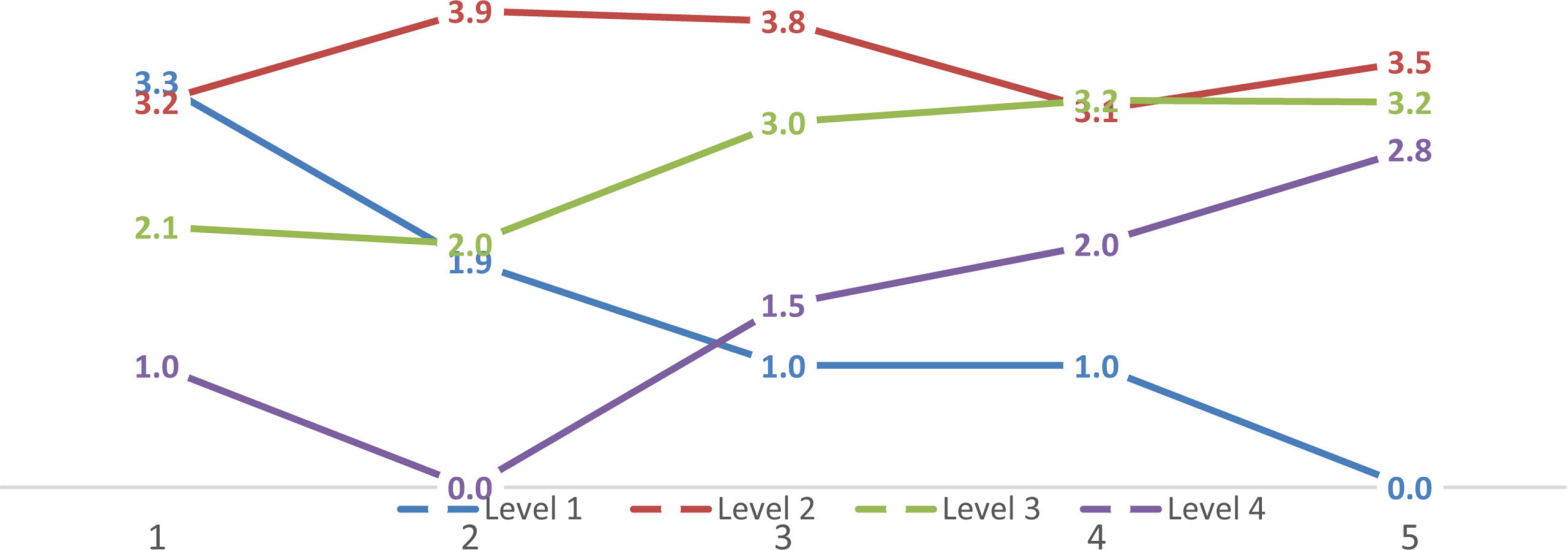
Number of group management facilitation skills per participant (mean) at each level.

The mean number of harmful behaviours per participant decreased from 3.3 at baseline to 1.9 post-training and further reduced to 0.0 during the supervision phase. **Figure 3** shows a continued decline in harmful behaviours during the supervision phase, alongside a notable increase in higher-level competencies, indicating that supervision contributed significantly to consolidating and advancing facilitation skills.

In parallel, the number of participants achieving Level 3 (all basic skills) and Level 4 (advanced skills) increased steadily throughout the supervision period, with a corresponding increase in mean scores from 2.0 to 6.3.

### 3.2 Implementation outcomes: self-reported confidence

**Figure 4:**
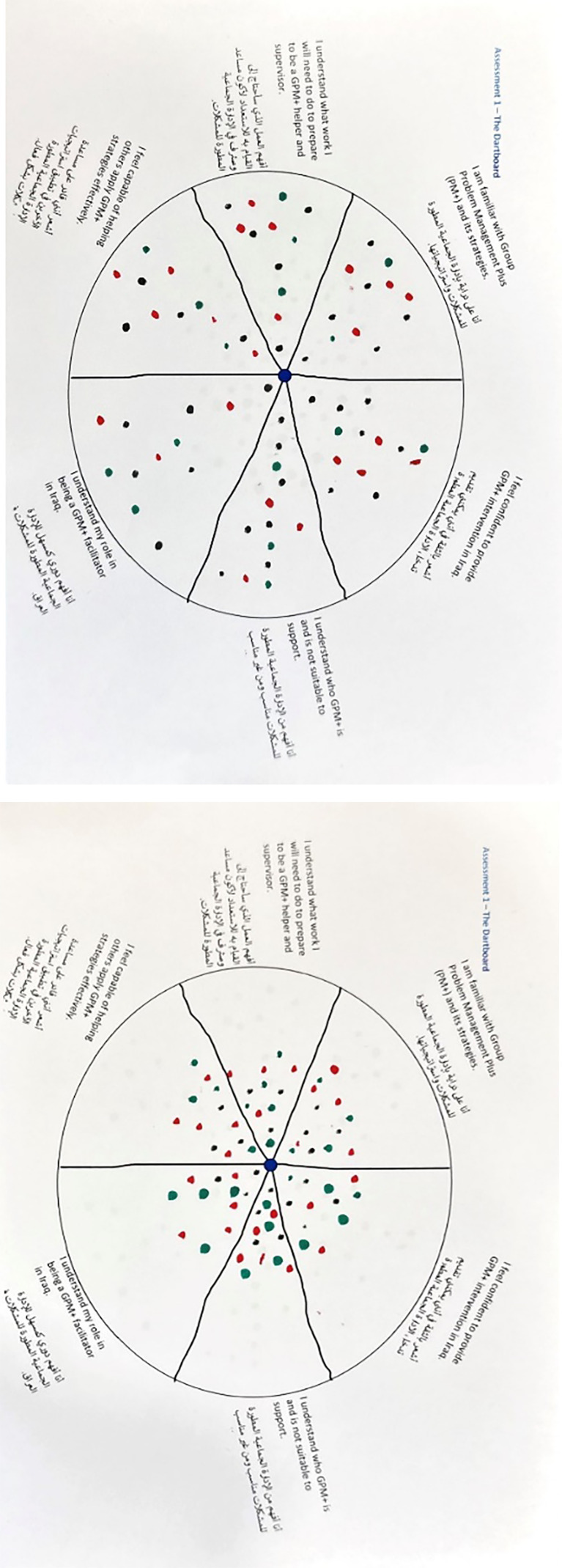
Self-efficacy as reported by participants.

In the pre-training assessment, the dots were widely distributed, indicating that participants had varying levels of confidence in their gPM+ knowledge and skills, with many feeling not very confident. As shown in **Figure 4**, the post-training distribution shifted noticeably toward the centre of the dartboard, indicating a clear overall increase in perceived confidence across competencies.

### 3.3 Implementation outcomes: acceptability of training

#### 3.3.1 Training evaluation results

Participants reported high levels of satisfaction with the training. Mean scores across all evaluation items ranged from 3.6 to 4.8, indicating generally positive perceptions of training content, delivery, and supervision (**Table 2**).

**Table 2:** Training evaluation results.

Training evaluation		
Question		Mean
1	The information was clear and easy to understand	4.0
2	The information I received is useful to my work.	4.8
3	The training increased my confidence to offer “Problem Management Plus – Group).	4.3
4	The training gave me practical skills and knowledge to apply in conflict situations.	4.1
5	The teaching methods used by the facilitator were effective.	4.0
6	The training was engaging and fun.	3.6
7	The facilitator created a supportive atmosphere in the training.	4.3
8	I would recommend this training to others.	4.4
9	The facilitator delivered the training to my expectation	4.2
10	The supervision sessions were effective, and I strongly recommend similar approach for upcoming trainings.	4.5

#### 3.3.2 Key findings from qualitative data

Participants identified several aspects of the training as particularly useful. The problem-solving strategies introduced during the gPM+ group sessions were widely valued, as they supported effective coping with challenges and enhanced resilience. The emphasis on breaking the cycle of inactivity was also reported as helpful, with participants noting that it provided practical tools to address procrastination and promote goal-directed behaviour. Techniques for managing psychological stress, particularly deep-breathing relaxation, were described as useful for coping with stress and anxiety. Participants also highlighted the opportunity to practise skills in group settings, which facilitated peer learning and reinforced practical relevance. The exchange of diverse experiences and perspectives within the group was also reported as beneficial for learning.

Some elements were perceived as less useful. While deep-breathing relaxation techniques were reported as helpful by certain participants, others found them difficult to apply in daily life. In addition, the strategy of rebuilding old relationships was often viewed unfavourably, with many participants expressing limited interest in this approach.

Participants offered several suggestions for improvement. They recommended shorter sessions delivered over more days to enhance engagement and retention, along with increased use of role-plays and practical exercises to strengthen skill application. They also requested clear action plans to support delivery of training content when implementing gPM+ in practice. Ensuring access to necessary materials for group sessions was identified as important, and some participants suggested incorporating topics and techniques addressing common adolescent issues in future training.

### 3.4 Trainer’s observations

Trainers reported that the EQUIP platform was a useful tool for completing assessments, identifying participant needs, and analysing data effectively. They noted that competency-based assessments can be challenging to implement, particularly when time is limited and there is insufficient capacity for rapid data analysis to inform immediate adaptations. However, the EQUIP platform streamlined the data collection process, enabled multiple assessors, and provided visual data analysis per group and per participant. Trainers reported that these features enabled them to tailor supervision sessions based on participants’ needs.

The platform allowed trainers to view overall competency scores alongside detailed information for each participant and each competency skill after completing the assessments. Trainers also reported that the platform was user-friendly and could be used by different assessors. When more than one trainer or supervisor conducted assessments for the same participant, the platform displayed the lowest score as the final score. Trainers also noted that the option to view the selected harmful or helpful behaviours helped identify priority skills for the upcoming supervision sessions.

## 4 Discussion

### 4.1 Training effectiveness

The training was associated with improvements in participants’ use of helpful skills and reductions in unhelpful or potentially harmful behaviours. Improvements were particularly evident during supervision sessions, suggesting that individual support may plays an important role in strengthening competencies. This underscores the importance of providing personalised feedback and tailored guidance alongside group-based training. The findings align with Elnasseh et al. (26), who emphasised that competency-based feedback delivered through structured platforms like EQUIP can support non-specialists’ confidence and ability to implement psychological interventions effectively.

In this study, the platform enabled trainers to identify specific areas for improvement for each participant, which appeared to contribute to the observed changes during training and supervision. Similarly, recent findings by Hemmo et al. (27) indicated that EQUIP tools can effectively identify individual strengths and areas for improvement while providing actionable feedback for trainers, highlighting their potential to enhance the overall quality of mental health care.

Additionally, the finding that competency improvements were most evident during supervision sessions is consistent with broader evidence suggesting that follow-up support is likely important for effective implementation in humanitarian settings (20). The results of this study also align with Böhm et al. (28), who emphasized that the use of simple, adaptable tools is essential in humanitarian settings, where time and resources are often limited.

While EQUIP was a valuable tool, trainers found the full assessment process time consuming. Simplifying the assessment and improving data handling may enhance its operational effectiveness, aligning with the need to streamline tools for field implementation. These operational challenges, including the time-intensive nature of competency assessments, reflect broader implementation barriers in which ‘protected time’ and adequate resource allocation are often required for effective supervision (19, 29). These observations further reflect implementation-level constraints, particularly related to workforce capacity and resource availability within the broader health system.

In this study, allowing participants to act as peer assessors during skill-focused role plays was a useful way to enhance engagement while also reducing trainer workload. This peer-driven learning approach aligns with the principle that successful capacity-building requires flexibility and practical tools (29).

Furthermore, the training reinforced the importance of adapting interventions to the local context, as emphasised by the STRENGTHS Consortium (43). Linguistic and dialectal variations, particularly between Kurdish dialects such as Sorani and Badini, were relevant during training delivery and role-plays. The trainer and co-facilitators were fluent in both dialects as well as English, which supported real-time clarification and culturally appropriate facilitation of competencies during practice sessions. However, the competency assessments were conducted exclusively in English using the EQUIP framework. This language discrepancy may have influenced how participants expressed competencies during role plays and how these were interpreted and scored by assessors, potentially affecting the sensitivity of the assessment to nuanced local expressions of competence. The effectiveness of the gPM+ intervention may have been supported by the adaptation of case studies, language, and training methods to the local cultural context. Trainers combined technical expertise with familiarity with both the content and the local context, which may have facilitated the learning experience and the integration of skills into practice. This suggests that contextual relevance plays a key role in successful implementation, aligning with the broader literature that advocates for cultural adaptation in the delivery of mental health interventions (22).

Additionally, the use of interactive activities, such as energisers and skill-building exercises like the juggling game, appear to support participants’ confidence and resilience. While not commonly referenced in existing literature, the juggling game and similar self-efficacy games proved to be valuable tools for demonstrating the concepts of gradual skill-building, perseverance, and self-efficacy. The positive impact of these activities on self-efficacy aligns with the conceptual framework presented by Wells et al. (19), which posits that clinical self-efficacy is a key mechanism through which supportive supervision improves practitioner well-being and service quality. These findings are also consistent with literature highlighting the importance of experiential and interactive methods for reinforcing skills and enhancing participant confidence (18).

These findings reflect improvements in provider-level competencies and should be interpreted as implementation outcomes rather than evidence of clinical effectiveness of the intervention.

From an implementation perspective, the findings can be further interpreted using the dimensions of the RE-AIM framework.The programme demonstrated initial reach and adoption through the engagement of DoH staff working across refugee camps and formal collaboration with the Directorate of Health. Implementation was largely feasible within the existing structure, with training and supervision delivered as planned, although constrained by time and resource limitations. However, maintenance and scale-up remain key challenges, particularly in light of funding reductions, the absence of trained supervisors within the DoH system, and the reduction of MHPSS service delivery in camps. While the programme was officially supported by the Directorate of Health and formed part of a broader strategy to integrate scalable psychological interventions into routine services; the plan could not be fully realised due to subsequent funding constraints, and the EQUIP platform was not formally institutionalised. The absence of trained supervisors within the DoH system and the reduction and closure of several MHPSS activities in camps due to humanitarian funding cuts have significantly limited continuation and scale-up.

These findings highlight the importance of aligning competency-based training approaches with broader system-level support to ensure sustainability and potential scale-up in similar humanitarian settings.

### 4.2 Challenges

Several challenges and limitations in the EQUIP platform were identified that may affect its usability, flexibility, and overall impact. One challenge was that assessors were required to complete the entire assessment questionnaire, covering both knowledge and skills, even when certain aspects were not practised during role-play exercises. This increased the workload. Another limitation was that results could only be extracted separately for each assessment, with no option to consolidate all data into a single, organised file for more comprehensive analysis. Finally, the platform’s analysis tools lacked interactivity and provided only basic insights. For example, while the number of participants displaying harmful behaviours slightly decreased from nine to eight, the more meaningful reduction in the mean number of harmful behaviours per participant from 5.6 to 2.0 was not readily highlighted by the platform. These challenges suggest the need for more user-friendly, interactive, and comprehensive features to maximise the platform’s effectiveness.

The implementation of the training programme was also influenced by several contextual factors that shaped both opportunities and challenges. High participant acceptance and strong collaboration with the Directorate of Health supported the delivery of the training, although motivation varied across participants. At the same time, structural constraints, particularly budget prioritisation, limited sustainability and scale-up. The absence of trained supervisors within the DoH system and the reduction of MHPSS services in refugee camps further constrained continuation. These findings highlight how system-level and individual-level factors influence the feasibility and sustainability of competency-based training in humanitarian settings.

### 4.3 Limitations

This report reflects findings from a training programme that was not initially embedded within a research framework. A structured evaluation design would have strengthened the analysis and allowed for more systematic assessment. While the training was adapted from an existing gPM+ curriculum, cultural and contextual modifications were limited by the standardised format, although this also supports comparability with other settings. In addition, the small sample size (n = 12) represents a significant limitation, restricting the generalisability of the findings and requiring cautious interpretation when applying results to other settings or populations. Furthermore, long-term outcomes were not assessed, reflecting concerns raised in a recent scoping review, which emphasised the need for sustained supervision and follow-up to ensure the long-term effectiveness of gPM+ interventions (44).

### 4.4 Future research

Future research should explore the effectiveness of competency tools in enhancing the skills of non-specialist MHPSS practitioners, particularly within scalable psychological interventions. It should also examine the role of supervision in supporting these practitioners, focusing on how different approaches to supervision influence practitioner development and client outcomes. Additionally, long-term studies are needed to assess the sustainability of skills acquired through competency-based training and how this may support the integration of MHPSS interventions developed for humanitarian settings into broader national health systems (45). This will likely require innovative partnerships among local and regional public health authorities, humanitarian and development agencies, and academic institutions (46).

For the EQUIP platform, future research could focus on its usability, the impact of supervision-related features, and the contribution of peer assessment during role plays to learning. Research should also investigate how the platform can be adapted to diverse cultural contexts, particularly for non-specialist practitioners across various settings. Furthermore, additional research is needed to examine the extent to which the additional costs associated with competency-based tools within the EQUIP platform lead to incremental quality gains and whether these investments are ultimately cost-effective, for which there are some early indications (47).

## 5 Conclusion

The training was associated with improvements in competency among non-specialist providers, particularly when supported by structured supervision. Notably, supervision sessions proved particularly impactful at the individual level, highlighting the importance of personalized support in driving meaningful change.

The EQUIP platform appears to be a useful tool for enhancing training delivery by providing detailed, actionable insights into participant progress at both individual and group levels. Its ability to track changes over time may support more targeted supervision and training adaptation.

To maximise the platform’s potential, simplifying the assessment process, improving data extraction and analysis capabilities, and integrating a digital trainer assessment feature would streamline operations and enhance training quality. With these refinements, the EQUIP platform may continue to support efforts to strengthen MHPSS training and interventions.

Overall, this training highlighted the importance of combining structured tools such as EQUIP with supervision, contextual adaptation, and interactive, practical learning. This integrated approach, particularly in humanitarian settings, has the potential to contribute to more effective and sustainable mental health interventions for non-specialists in low-resource environments.

From an implementation perspective, the programme engaged DoH staff working in refugee camps, although this represented a relatively small proportion of the overall workforce. Sustainability and scale-up remain key challenges, as supervision was only partially maintained and the absence of trained PM+ supervisors within the DoH system, combined with funding reductions affecting both UNHCR and public health services, constrained continuation of MHPSS activities.

This study contributes real-world implementation evidence on competency-based training using EQUIP in a humanitarian setting, highlighting feasibility and skill development rather than causal effectiveness.

## Data Availability

The data analyzed in this study is subject to the following licenses/restrictions: the data is available on EQUIP platform and access can be granted upon request. However, the data cannot be easily extracted in an excel sheet (or any other format), which is one of the mentioned challenges of using the platform. Requests to access these datasets should be directed to Tekoshar Aram, tekoshar.aram@koyauniversity.org.
